# Lattice-based PKEs/KEMs

**DOI:** 10.1093/nsr/nwab090

**Published:** 2021-05-24

**Authors:** Xianhui Lu, Jiang Zhang

**Affiliations:** Institute of Information Engineering, Chinese Academy of Sciences, China; State Key Laboratory of Cryptology, China

The invention of public-key cryptography (PKC) by Diffie and Hellman in 1976 is one of two milestones marking the beginning of modern cryptography. The security of a PKC system requires that it should be infeasible to compute the private key from a given public key, which in turn is typically guaranteed by the difficulty in solving some cryptographic-friendly mathematical problems. Among those problems, the integer factorization and discrete logarithm problems play pivotal roles in the development of public-key cryptography. In particular, the assumption that there is no polynomial time (classical) algorithm that solves the above two problems constitutes the basis for the security of almost all currently used public-key cryptosystems, such as RSA and ElGamal. However, Shor [1] found an efficient quantum solving algorithm for the integer factorization and discrete logarithm problems in 1994, which would destroy the security basis for most real deployed PKC systems if large-scale quantum computers become available. The rapid development of quantum technology in recent years suggests that we are getting closer to the quantum crisis of current PKC systems.

As a response to the quantum crisis, the research community has made many efforts to post-quantum cryptography (PQC) that are believed to resist quantum computer attacks. Lattice-based cryptography is one of the main directions in this area, and has become the most promising PQC candidate for standardization.

The research of lattice-based cryptography dates back to the seminal work of Ajtai [2], which first based the security of cryptographic primitives on the difficulty of solving some lattice problems such as the approximate shortest vector problem and the closest vector problem. After more than two decades of development, lattice-based cryptography has gained substantial progress. In theory, we have witnessed the construction of many powerful cryptographic primitives that were not known before. In practice, the public key size of lattice-based cryptosystems has been significantly reduced from several gigabytes to a few kilobytes.

For current applications and standardization, key exchanges, public-key encryptions (PKEs) and signatures are the most desired lattice-based cryptosystems, where the first two are often used to ensure information secrecy, while the last provides information authentication. Note that public-key encryptions are equivalent to two-round key exchanges, also known as the key encapsulation mechanism (KEM). In the following, we focus on lattice-based PKEs/KEMs.

The design principle of lattice-based PKEs/KEMs follows two approaches: a trapdoor one-way function and an approximate commutative one-way function. In the first approach, a trapdoor one-way function *f* and its trapdoor *f*^−1^ are generated as the public key and private key. A plaintext *m* is encrypted as *c* = *f*(*m*), and the ciphertext is decrypted as *m* = *f*^−1^(*c*). The NTRU scheme and its variants follow such an approach. In the second approach, an approximate commutative function *f*_*s*_ and a random input *a* are generated, the public key is (*a*, *b* = *f*_*s*_(*a*)) and the private key is *s*. A plaintext *m* is encrypted as *c*_1_ = *g*_*r*_(*a*), *c*_2_ = *g*_*r*_(*b*) + *E*(*m*), where *r* is a random element, *g* is an approximate commutative one-way function and *E* is the encoding of an error-correction code. The ciphertext (*c*_1_, *c*_2_) is decrypted as *m* = *D*(*c*_2_ − *f*_*s*_(*c*_1_)), where *D* is the decoding of an error-correction code. The correctness of decryption is guaranteed by the approximate commutative property (*g*_*r*_(*b*) = *g*_*r*_(*f*_*s*_(*a*)) ≈ *f*_*s*_(*g*_*r*_(*a*)) = *f*_*s*_(*c*_1_)) and the error-correction code. Learning with errors (LWE) problem-based PKE/KEM schemes and their variants follow such an approach.

The standardization of PQC has received substantial support from national funding agencies, such as the European Union projects PQCrypto and SAFEcrypto. The USA National Institute of Standard and Technology (NIST) started the post-quantum standardization project in 2012, organized a worldwide ‘competition’ in 2016 and plans to deliver draft standards before 2024. After two rounds of evaluation, seven schemes were accepted as the round-three candidates on 22 July 2020, of which five are lattice-based schemes. In 2017, the International organization for standardization started a study project of post-quantum cryptography named SD8.

Most LWE-based PKE schemes such as Kyber and LAC follow the Lindner-Peikert framework [3] up to the choices of ring structure, noise distribution and parameters. As the size of noise in lattice-based PKEs/EKMs is typically larger than the lower bits of the ciphertext, many schemes often artificially drop a few lower bits to optimize the practical performance without significantly increasing the final decryption noise.

Kyber [4] is one of the most promising PKE/KEM candidates in the NIST PQC standardization, whose security is provably based on the difficulty of so-called module LWE (MLWE) problems. The use of MLWE allowed Kyber to obtain additional flexibility and security advantages, while keeping the same efficiency as those based on the ring-LWE problems. The strategy of dropping some lower bits is used to compress both the public-key and ciphertext sizes. For a targeted 128-bit quantum security, the public-key and ciphertext sizes of Kyber is around a single kilobyte.

LAC [5] uses several techniques to reduce the modulus as small as possible: it uses a byte-level modulus, embeds powerful error-correcting codes to maximize the noise-modulus rate, provides a different style of modulus to avoid potential attacks for special polynomial ring structures and optimizes the polynomial multiplication algorithm of the bit-level secret and noise. Based on these techniques, LAC reduces the public key to 544–1056 bytes and the ciphertext to 664–1464 bytes. Meanwhile, it achieves similar computational performance compared with NTT technique schemes.

By observing the asymmetries in lattice-based cryptosystems, Zhang and Yu *et al.* [6] proposed asymmetric MLWE and asymmetric MSIS problems, which yield further size-optimized KEMs and signature schemes to those from standard counterparts. Compared to the seven NIST round-three candidates at Category III security, their KEM scheme has parameters (public key + ciphertext size) smaller than Classic McEliece, CRYSTALS-Kyber, NTRU and Saber; their signature scheme has parameters (public key + signature size) smaller than CRYSTALS-Dilithium and Rainbow, but slightly larger than FALCON, where CRYSTALS-Kyber, NTRU, Saber, CRYSTALS-Dilithium and FALCON are lattice-based schemes.

One important application of lattice-based PKEs/KEMs is to build authenticated key exchange (AKE), which could provide additional authentication to the established session key. There are several generic ways to construct AKE from lattice-based PKEs/KEMs. In the PKE setting, Xue and Lu *et al.* [7] proposed a more compact KEM-based framework via a new primitive named ‘2-key KEM’, which shares some item between different ciphertexts. In the only password setting, Zhang and Yu [8] presented a two-round PAKE framework from the splittable-PKEs that can be instantiated from lattices. The only known AKE that does not rely on lattice-based PKEs/KEMs was given in Ref. [9], which has two-round messages and provides implicit authentication.
